# Alcohol consumption impairs the ependymal cilia motility in the brain ventricles

**DOI:** 10.1038/s41598-017-13947-3

**Published:** 2017-10-20

**Authors:** Alzahra J. Al Omran, Hannah C. Saternos, Yusuf S. Althobaiti, Alexander Wisner, Youssef Sari, Surya M. Nauli, Wissam A. AbouAlaiwi

**Affiliations:** 1 University of Toledo, College of Pharmacy and Pharmaceutical Sciences, Department of Pharmacology and Experimental Therapeutics, Toledo, Ohio USA; 2 Taif University, College of Pharmacy, Department of Pharmacology and Toxicology, Taif, Kingdom of Saudi Arabia; 3 Chapman University, College of Pharmacy, Irvine, California USA

## Abstract

Ependymal cilia protrude into the central canal of the brain ventricles and spinal cord to circulate the cerebral spinal fluid (CSF). Ependymal cilia dysfunction can hinder the movement of CSF leading to an abnormal accumulation of CSF within the brain known as hydrocephalus. Although the etiology of hydrocephalus was studied before, the effects of ethanol ingestion on ependymal cilia function have not been investigated *in vivo*. Here, we report three distinct types of ependymal cilia, type-I, type-II and type-III classified based upon their beating frequency, their beating angle, and their distinct localization within the mouse brain-lateral ventricle. Our studies show for the first time that oral gavage of ethanol decreased the beating frequency of all three types of ependymal cilia in both the third and the lateral rat brain ventricles *in vivo*. Furthermore, we show for the first time that hydin, a hydrocephalus-inducing gene product whose mutation impairs ciliary motility, and polycystin-2, whose ablation is associated with hydrocephalus are colocalized to the ependymal cilia. Thus, our studies reinforce the presence of three types of ependymal cilia in the brain ventricles and demonstrate the involvement of ethanol as a risk factor for the impairment of ependymal cilia motility in the brain.

## Introduction

The motile ciliated cells are ubiquitous in the lung airways, the fallopian tubes in the uterus, the efferent ducts in the testes and sperm tail, as well as in the brain ventricles and spinal cord canal. Primary ciliary dyskinesia (PCD), male infertility and hydrocephalus are phenotypes that result from motile cilia dysfunction. Hydrocephalus is a combination of neurological and neuropathological manifestations characterized by an imbalance between the production and absorption of CSF. This disparity leads to an extreme accumulation of fluid causing the brain ventricular cavities and the subarachnoid spaces to expand in response to the increase in CSF^1,2^. Although hydrocephalic symptoms are constant, the disease is classified into two types based upon the etiology- congenital and non-congenital. Congenital, also known as neonatal hydrocephalus occurs due to genetic abnormalities in combination with other conditions such as intraventricular hemorrhage and/or ethanol abuse during pregnancy^3–7^. Non-congenital hydrocephalus occurs after birth resulting from an injury or infection of the brain ependyma^2,8,9^.

The ependymal cilia help distribute and circulate the CSF from its origins in the choroid plexus located in the lateral and the third ventricles. The CSF flows in a unilateral direction starting in the lateral ventricle, then moving through the third ventricle, and then the fourth ventricle where it passes into the subarachnoid space insulating the brain and spinal cord. The direction of flow depends upon the ependymal cellular orientation, which in turn is dependent upon the motile cilia^2,10,11^.

The motile cilia dynamics deteriorate in hydrocephalic conditions for several reasons. First, any mutation within the essential proteins responsible for ciliogenesis or cilia ultrastructure will lead to a disruption in cilia motility^12^. There are several markers for the motile cilia, each localizing to specific areas. Either a mutation in one of these protein markers or an altered localization is associated with motile cilia dysfunction^13^. *Hydin* is a hydrocephalous-inducing gene that encodes for a central pair protein within the axoneme of motile cilia. A mutation in *hydin* leads to dislocation and eventual loss of the central pair, which disturbs the movement of cilia leading to hydrocephalous^14,15^. *Hy3/hy3* mutant mice show early hydrocephalous onset and an enlargement of the lateral and third ventricles, as a result of abnormal CSF flow caused by the impairment of cilia movement. The beating frequency of ependymal cilia in the *hydin* mutant mice declines significantly due to changes in the location of the central pair microtubules leading to ineffective fluid movement^13^.

Polycystin-2 (PC-2) is a membrane-associated protein known to be expressed in primary cilia and plays an important role in the mechanosensory and ciliogenesis functions of motile cilia^16^. Polycystin-2 is also associated with sperm development and motility. *Pkd2* mutant Drosophila develops male sterility as a result of declining sperm movement^17^. Polycystin-2 serves as a sensory protein that detects changes in fluid flow within the respiratory ciliated cells and contributes to the development of brain planar cell polarity (PCP) and prevention of hydrocephalus^18^.

Ethanol consumption has been reported to reduce respiratory performance due to its significant effect on mucociliary clearance. Recent evidence suggest a dose-dependent relationship between alcohol use and thinner cortex and ventricular expansion even at lower alcohol concentrations and in healthy individuals as well^19^. Previous studies from our lab had investigated the dynamics of ependymal cilia in the third ventricle and found a decrease in beating among the cilia within the third ventricle after ethanol incubation *ex vivo* ^20^. In the current study, we show, through cilia beating frequency and beating angle, that ependymal cells in the brain lateral ventricle can be distinctly categorized into three types with each type uniquely localized in the ventricle. Furthermore, we show for the first time that oral gavage of ethanol alters the beating of ependymal cilia in the rat brain lateral and third ventricles. Thus, our studies suggest a direct link between ethanol dependence and hydrocephalus involving ependymal cilia function.

## Clinical Implications and Significance

Dysfunctional cilia have been associated with a large number of diseases characterized by pleiotropic symptoms collectively referred to as “ciliopathies”. Hydrocephalus is a ciliopathy characterized by CSF accumulation in the brain caused by genetic mutations that lead to impairment of ciliary motility. Variations in ciliary motility can be influenced by changes in ciliary beating patterns as well as ciliary beating frequencies, both of which correlate with the patients’ genotype. Hence characterizing the different types of ependymal cilia based on variations in ciliary motility may provide a guide for identifying the mechanism responsible for ciliopathies in the brain. This is the first report to demonstrate the involvement of ethanol as a risk factor for cilia motility impairment in the brain.

Several factors are responsible for causing hydrocephalus such as brain injury, infection, or genetic factors coupled with a secondary stressor. A common secondary stressor is maternal alcohol consumption while pregnant; research on brain abnormalities have shown hydrocephalic symptoms in some offspring prenatally exposed to alcohol^3^. However, despite a wealth of literature, the mechanism linking alcohol consumption to hydrocephalus remains unknown. Treatment for hydrocephalus is not started until the child is born and typically requires surgery to alleviate the symptoms. Unfortunately, surgical intervention is not always effective long-term^4,21^. Thus, elucidating the mechanism of hydrocephalus pathophysiology may provide the necessary information to develop more effective pharmacological compounds that could target cilia motility as a therapeutic option^22^.

## Results

### Live imaging of ependymal cilia

**Figure 1:**
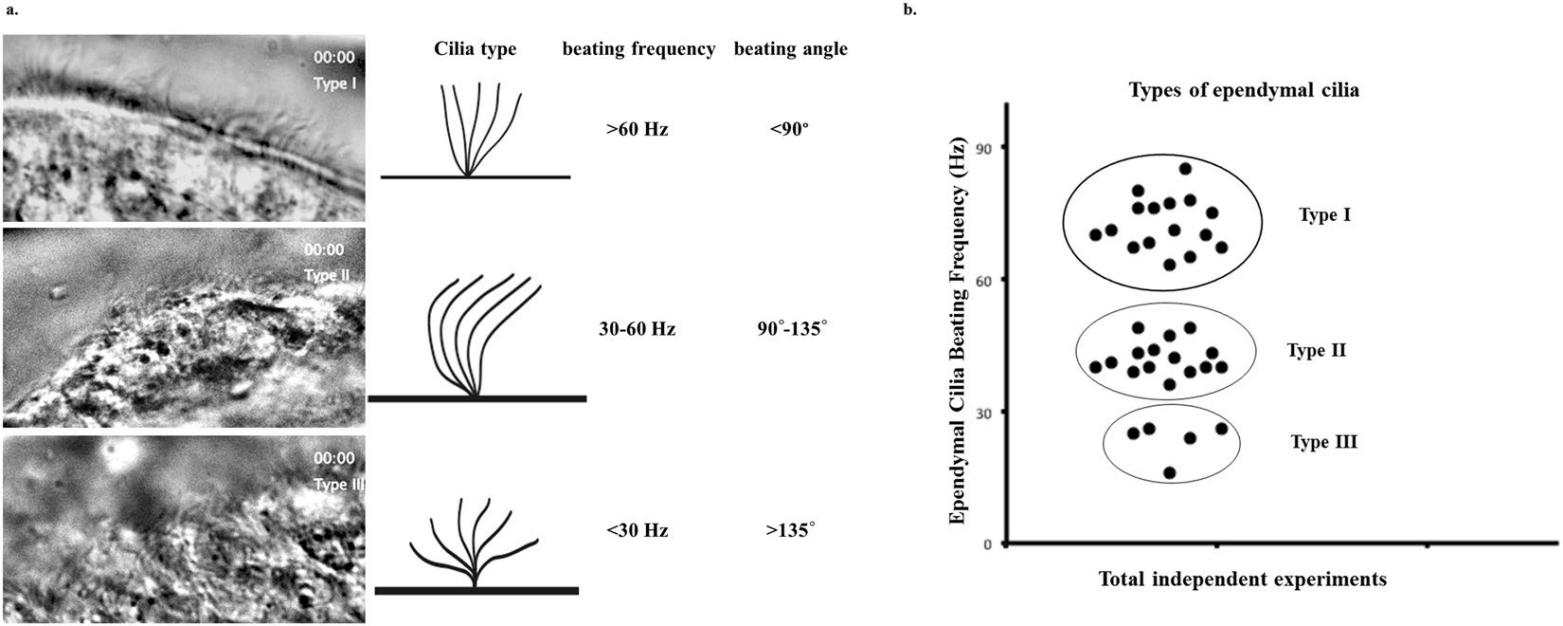
Ependymal cilia of the brain lateral ventricle are classified into three types based on their beating frequency and angle. (**a**) Shown here are representative DIC images of the ependymal cells of the mouse brain lateral ventricle with the three types of motile cilia. The images of the ependymal cilia are taken from a time-lapse movie during a live imaging experiment. (**b**) Classification of ependymal cilia is based upon their beating frequency and angle. Each dot represents an independent experiment. Type I cilia are the fastest and has a beating frequency >60 Hz with a beating angle less than 90°. Type II beating frequency is between 30–60 Hz with a beating angle between 90°–135°. Type III cilia have the slowest beating frequency <30 Hz and a beating angle >135°.

We developed a novel technique to allow close observation of the movement of ependymal cilia within the brain ventricles. By obtaining a sagittal plane section from the mouse brain, we can expose the ependymal cilia with minimum reduction in vitality and image quality by using a high-resolution differential interference contrast (DIC) microscopy system producing live images and movies (Fig. 1a). A step-by-step procedure detailing the sectioning, imaging, and staining of our brain sections was published recently^23^.

### Ependymal cilia are classified into three types based on their beating frequency and angle

**Figure 2:**
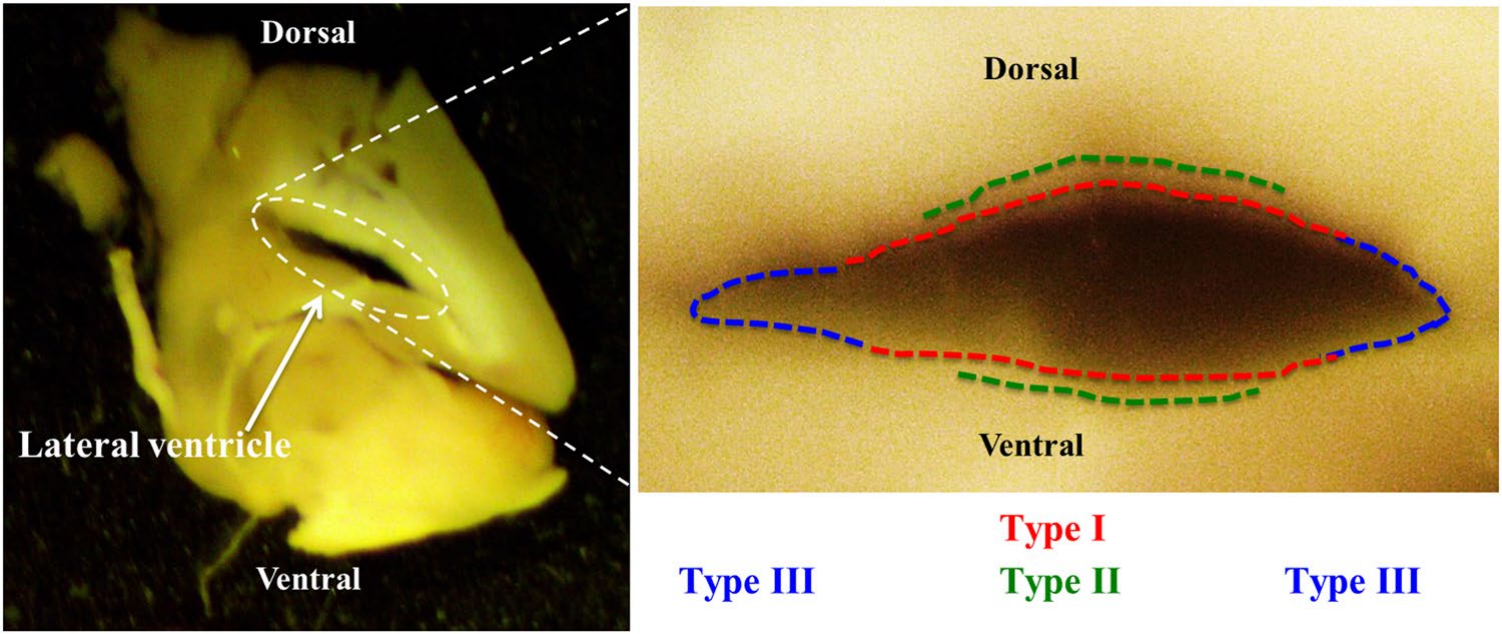
Each type of ependymal cilia has specific localization within the brain lateral ventricle. This figure shows a sagittal section view of the lateral ventricle (left). Each type of the ependymal cilia (shown on the enlarged area) is localized within specific area in the lateral ventricle based on the beating frequencies and angle of movement.

The live movie analysis of the cilia beating in the lateral ventricle revealed remarkable variations in the beating frequencies. This enabled us to classify the cilia into three distinct types with regards to their beating frequency, movement angle and pattern (Fig. 1b). Type I cilia beat the fastest at >60 Hz and have the least volume replacement stroke with a beating angle of <90° (Movie 1). The beating frequency of type II cilia was 30–60 Hz with a beating angle between 90° and 135° (Movie 2), while type III cilia have the slowest beating frequency of <30 Hz but the largest volume replacement stroke with a beating angle of >135° (Movie 3). The ciliary beating in the lateral ventricle mimics that of the cilia within the third ventricle^20^. It is worth mentioning that the three types of ependymal cilia are located in specific locations within the lateral ventricle. We mapped the distributions of ependymal cell types within the lateral ventricle. Following our mapping analysis, we demonstrated that type I cells are mostly distributed along the ventricle dorsal and ventral walls, but they are absent from both corners of the lateral ventricle. Type II cells are mainly distributed in the middle of the dorsal/upper wall of the ventricle, but they could also be found in the middle of the ventral/lower wall of the ventricle. Type III cells are distributed almost exclusively at the corners of the lateral ventricle (Fig. 2).

### *Ex-vivo* exposure to ethanol decreases ependymal cilia beating in the brain lateral ventricle

**Figure 3:**
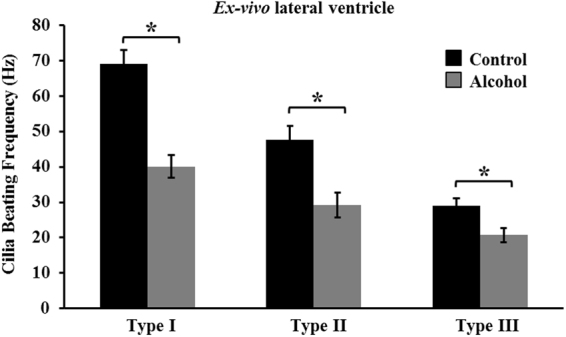
Ethanol decreased cilia beating *ex vivo*. Treatment of *ex vivo* brain slices with 0.25% ethanol for five minutes shows a significant reduction in the beating frequency of all three types of ependymal cilia. Up to 22 independent preparations were used and the presence of an asterisk (*) denotes significant difference at p < 0.05.

The *ex-vivo* ethanol experiment was performed to examine the accuracy of the ependymal cell classification, as well as to investigate whether the effect of ethanol is consistent within all three types of ependymal cilia. In addition, it enabled close observation of the effect of oral gavage of ethanol on the ependymal cilia in controlled environmental settings. Data from the *ex-vivo* experiments indicate that there is a significant decline in cilia motility after incubating the brain sections in 0.25% ethanol for 5 minutes. By analyzing and comparing the movement of the three types of cilia before and after the ethanol treatment, we found a decrease in cilia movement occurring in all three types (Fig. 3 and Movies 3–6).

### Ethanol drinking decreases ependymal cilia beating in the brain lateral and third ventricles *in vivo*

**Figure 4:**
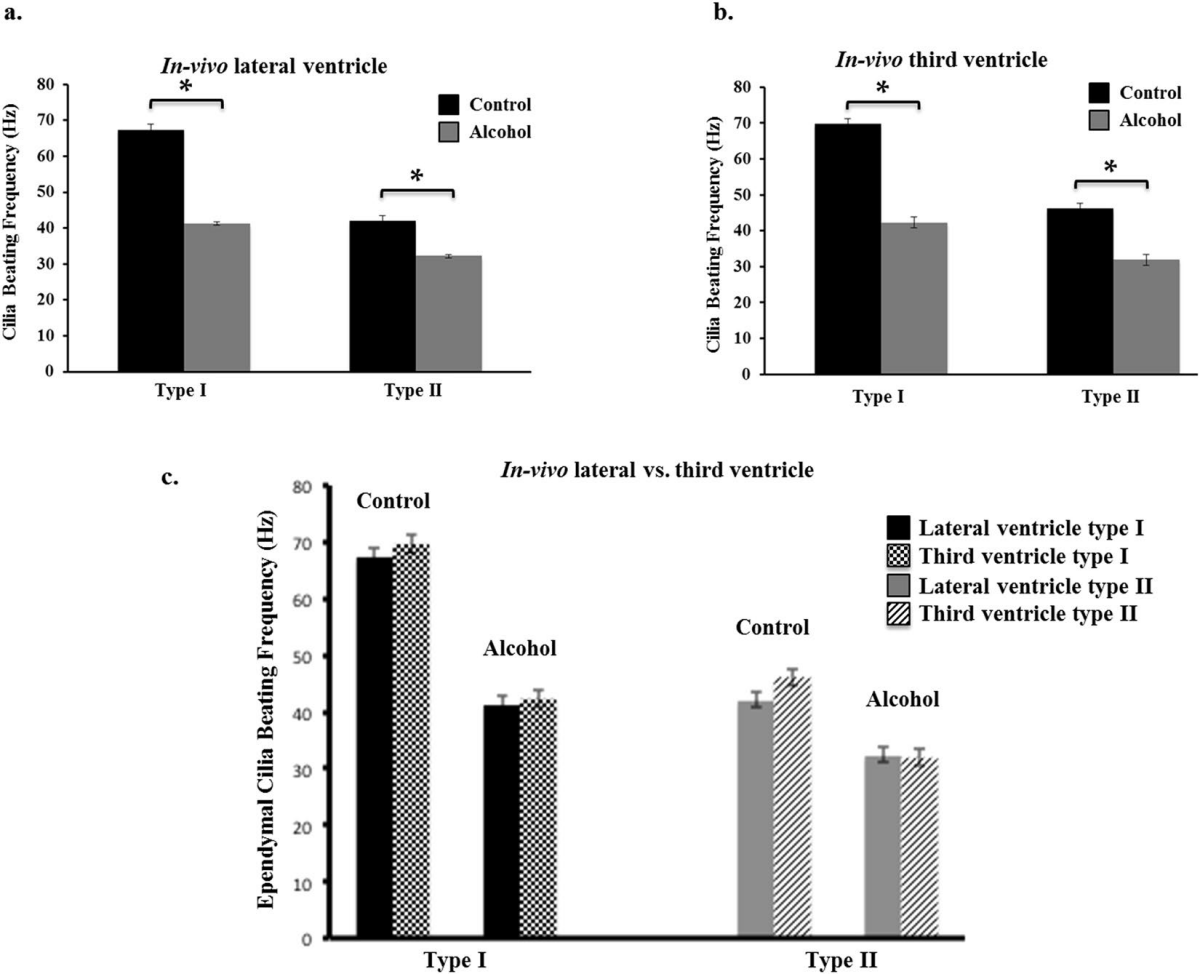
Alcohol drinking altered the dynamics of ependymal cilia in the brain. Two-month old Wistar rats were given either water or 95% ethanol (alcohol) at a 6 g per kg of body weight for seven days. After acute oral treatment with alcohol, the rat’s brain was dissected to examine the dynamics of ependymal cilia in both the lateral and third ventricles. Alcohol drinking caused a significant decrease in the beating frequency of the ependymal cilia of both, (**a**) the lateral and (**b**) third ventricles compared to the control group. (**c**) Further **c**omparison within the control groups and the alcohol-drinking groups between the dynamics of the ependymal cilia in the lateral ventricle vs. the third ventricle demonstrated no significant difference between the ciliary beating frequencies.

To confirm the physiological relevance of the *ex vivo* ethanol treatments, the effect of oral gavage of ethanol on ciliary beating frequency and ependymal cilia function were further investigated. Our data on ependymal cilia motility clearly demonstrated a significant decrease in the ependymal cilia dynamics after acute oral gavage of ethanol, which is supported by our previous findings (Fig. 4a,b). Studies into both the lateral and the third ventricles confirmed that cilia motility was affected by oral gavage of ethanol in the same manner (Fig. 4c and Movies 7–14).

### Hydin and polycystin-2 are expressed in the ependymal cilia

To verify our high-resolution differential interference contrast and fluorescence microscope systems, we examined the presence of ependymal cilia in the lateral ventricle. Ependymal cilia were confirmed with a ciliary marker, acetylated-α-tubulin (Supplementary Figure S2). In an effort to explore the mechanism or the structural differences that could explain the variation in movement and beating patterns among the three types of cilia, the ciliary localization of several key structural proteins were investigated for the first time by immunofluorescence staining. Hydin, a central ciliary axonemal protein, presented as a good marker to distinguish between the three types of cilia since it is located in the central pair of the motile cilia. Eventually, we were able to localize hydin in the ependymal cilia for the first time by immunofluorescence microscopy in the lateral ventricle. Hydin’s ciliary localization was confirmed in the lateral and third ventricles by co-staining with the ciliary marker acetylated-α-tubulin (Supplementary Figures S1 and S2). Polycystin-2′s known expression in airway motile cilia lead to the hypothesis that it may also localize to the motile cilia of the ependymal cilia^18^. Data from our immunofluorescence experiments demonstrated the localization of polycystin-2 to the cilia in the lateral ventricle (Supplementary Figure 6c). Hydin and Polycystin-2 were localized in all of the three types of the ependymal cilia. Future studies are warranted to examine other motile cilia proteins, which may be specific to each type of cilia such as CCDC115, CCDC114, and ARMC4 that encode for outer dynein arm proteins^24^.

## Discussion

We report, for the first time, the classification of ependymal cilia in the mouse brain lateral ventricle and confirmed the previous classification of the ependymal cilia in the third ventricle^20^. The ependymal cilia are divided among three distinct types in regard to their beating frequency and beating angle. Type I has a beating frequency greater than 60 Hz and a movement angle domain of less than 90°, type II has a beating frequency between 30–60 Hz and an angle between 90°–135°, and type III has a beating frequency less than 30 Hz and an angle greater than 135°. Although the three types vary in velocity and fluid volume movement due to differences in the beat patterns and speed, the three types of cilia are distributed equally throughout the lateral ventricle to maintain efficient circulation of the CSF. Our pharmacological screening confirms that *ex vivo* treatment of brain slices with 0.25% ethanol resulted in a reduced cilium beating frequency thus contributing to a decrease in the velocity and volume of fluid movement. More importantly, our studies show that ethanol ingestion in rats leads to an impairment of ependymal cilia beating and function in both the lateral and third brain ventricles *in vivo*. To our knowledge, this is the first report to study the *in vivo* effect of oral gavage of ethanol on the physiology of ependymal cells in the brain lateral and third ventricles.

Several studies introduce advanced methods for performing and analyzing high speed digital imaging of ependymal cilia^25,26^; however, classification of ependymal cells has not been reported before. To date, studies have indicated chemicals such as ethanol and its metabolite, acetaldehyde, have no effect on motile cilia beating frequency in concentrations between 0.1% and 1% ^27,28^. However, based on our new ependymal cilia classification parameters, *ex-vivo* studies on treated ependymal cell tissue, using a minimum concentration of 0.25% ethanol, reveal that ethanol causes a significant decrease in the ciliary beating frequency.

As previously mentioned, disruption in ependymal cilia function is one of the main reasons for the ventricle enlargement observed in hydrocephalous. Chronic ethanol consumption leads to numerous destructive effects on the brain, including an increase in the size of the brain ventricles by 31–71%, the hallmark of hydrocephalus^29,30^. Several studies have been undertaken to investigate the effects of ethanol on the brain in cases of chronic alcoholism; however, none have focused on the ependymal cilia in particular. In our study, we confirmed our findings of a reduction in ciliary beating frequency from the *ex-vivo* ethanol treatment. We also found, for the first time, that ciliary beating frequency *in-vivo* was significantly reduced, triggering a decrease in ciliary performance. This could potentially provide an explanation for the occurrence of hydrocephalus in patients suffering from acute alcoholism. Previous studies examining the effects of ethanol drinking and other toxic agents on motile cilia function have been mainly performed either on excised tissue or on primary ciliated epithelial cells grown directly from fresh tissue^31,32^. However, none of these studies examined the effect of ethanol consumption directly on the motility and function of ependymal cilia in the brain ventricles. It has been postulated that exposure of the airway epithelium to volatized ethanol from the bronchial circulation initially leads to a rapid increase in cilia beat frequency followed by a desensitization of ciliary stimulatory response^32^. Our studies reinforce the deleterious effects of ethanol consumption on the sensory function of ependymal cilia and provide a potential explanation for the brain-related symptoms that are associated with ethanol drinking such as headaches, difficulty walking, blurred vision, slurred speech, slowed reaction times, and others.

It is well established that cilia are specialized sensory compartments on the apical surface of most cells to sense and transmit information from the extracellular matrix to the cell interior. To perform its unique sensory roles, a high density of specialized proteins, such as receptors, ion channels, and other signaling modules localize in the ciliary compartment^33^. As reported in this study, our classification of ependymal cilia is based on differences within the movement patterns and velocity of beating. In an attempt to explain the variation in beating frequencies and angles with regards to the ultrastructure of the three types of motile cilia, we used antibodies against different proteins that are linked to the ciliary membrane or that recognize posttranslational modifications of ciliary axonemal molecules that can be used to differentiate between different types of motile cilia. In our study, we used ciliary markers that could potentially help us identify the unique pattern of different motile cilia types or shed light on the function of these ciliary markers.

Hydin is a central axonemal microtubule protein required for ciliary motility. Although the morphology of ependymal cilia in the brains of mutant animals is normal, one of the two central microtubules lacks a specific projection and the cilia are unable to bend normally, ciliary beat frequency is reduced, and the cilia tend to stall. As a result, these cilia are impaired and incapable of generating fluid flow. Thus, causing the phenotypes associated with hydrocephalus such as fluid accumulation in the brain^13^. Although *hydin* mutation has been shown to be directly associated with the pathology of hydrocephalus, to our knowledge, there has been no report showing the localization of hydin to the ependymal cilia in the brain. We were able to localize hydin for the first time throughout the entire axoneme of the cilia. Localization of hydin in the brain ventricle could assist in the diagnosis and the treatment of hydrocephalus^34^.

Polycystic kidney disease 1 (*Pkd1*) and *Pkd2* genes encoding for polycystin-1 (PC-1) and polycystin-2 (PC-2) form a mechanosensory complex in the primary cilia of kidney and vascular endothelial cells^35–37^. The mechanosensory proteins are recently shown to be present in primary cilia of radial glia cells (RGCs). Deletion of *Pkd1* or *Pkd2* in central and peripheral nervous system neuronal and glial cell precursors affected planar cell polarity (PCP) and development in RGCs and ependymal cells^38^. This study suggested that PC-1 and PC-2 mechanosensory proteins contribute to the brain development, PCP, and hydrocephalus prevention. Here, we further show that polycystin-2 is localized and detected in all three types of ependymal cilia in the brain ventricles. An understanding of the molecular mechanism leading to the pathogenesis of hydrocephalus, which could potentially vary by location of the ependymal cilia type, could help develop genetic tools for the diagnosis and treatment of hydrocephalus. This study identifies cellular and molecular components that could mediate hydrocephalus formation in ependymal cells.

## Methods

The Institutional Animal Care and Use Committee (IACUC) of The University of Toledo approved all of the procedures for animal use in accordance with the guidelines of the Institutional Animal Care and Use Committee at the National Institutes of Health and the Guide for the Care and Use of Laboratory Animals.

### Live imaging

For ependymal cilia live imaging, wild type mice strain C57BL/6 were euthanized by asphyxiation using a CO_2_ gas chamber for five minutes. Cervical dislocation was used as a second method of euthanasia to confirm death. A craniotomy was performed to collect the whole brain. A thin sagittal plane section was obtained and immediately placed in a glass-bottom petri dish containing 37 °C Dulbecco’s Modified Eagle Medium (DMEM)/High-Glucose (HyClone Inc.), 10% fetal bovine serum (FBS) (Fisher Scientific Inc.) and 1% penicillin/streptomycin solution containing 10,000 units/mL of penicillin and 10,000 µg/mL of streptomycin. To provide the fresh live tissue section with the proper environmental conditions to survive, the microscope’s enclosed chamber temperature has been adjusted to 37 °C with a gas mixture of 95%/5% O_2_/CO_2_. The cilia movement was recorded using an eclipse TE2000 microscope equipped with a 60x objective oil immersion lens and a differential interference contrast filter. Time-lapse images were captured and analyzed using Metamorph imaging software at a 1 × 1 binning with a 5–10 msec exposure time with at least 200 frames per second.

### Beating frequency measurement

After recording the live cilia movement, each movie was analyzed by counting the number of cilia beats per one second. The motile cilia fluid dynamics have two stages, the power stroke where the cilia arc becoming horizontal to the cell surface, and the recovery stroke where the cilia reneges its initial upright position. The power and recovery stroke of ependymal cilia are counted as one complete beat cycle. In order to accurately estimate the number of beats, the time-lapse movie may be slowed to a manageable speed while using a cell counter to count the number of beatings. Thus number of cilia beatings is counted in one minute using a cell counter and the frequency of beatings is calculated by multiplying the exposure time at which the video is recorded by the number of frames or time-lapse images acquired to get the number of sec. (Example: exposure time 5 msec. 200 frames = 1,000 msec or 1 sec). The number of cilia beatings over a one-second time interval (Example: cilia beats 50 times in a 200 frames video recorded at exposure time of 5 msec *i*.*e*. 5 msec × 200 frames = 1 sec; now divide 50 beats by 1 sec = 50 Hz). The ciliary beating angle is then calculated by evaluating the path taken by the ependymal cilia during both the power and recovery strokes. This is performed according to a previously described method, with minor modifications^23^. Briefly, a horizontal line along the ependymal edge and a vertical line through the midline position of the cilia at the start of the power stroke were drawn on an acetate sheet placed over the monitor. The position of the cilium is then plotted frame by frame as it moves forward during both the power and the recovery strokes. Finally, the ciliary beating angle is calculated from the maximum deviation of the cilium from the midline during the power stroke as well as the recovery stroke. At least 5–15 mice were used in each experiment and up to 20 preparations were counted from each mouse brain.

### Determination of different types of cilia within the lateral ventricle

To determine the specific location of each type of cilia and their relation to each other, a video for each type was recorded, marking the region of each video within the ventricle. To determine the type of cilia, after recording and analyzing the videos, the number of beats per second was counted and the angle of movement was measured. Subsequently a map showing the distribution of the cilia types in the ventricle walls is drawn.

### *Ex-Vivo* ethanol treatment

After dissection, a thin sagittal plane section of the mouse brain was obtained and incubated in 1 ml 37 °C DMEM/High-Glucose media. Then, the videos of the beating cilia were captured at an exposure rate of 5 milliseconds for at least 200 frames per second. To investigate the effect of ethanol in the ependymal cilia, the same section was incubated in 0.25% ethanol (190-proof, Decon Lab Inc.) in 37 °C DMEM /High-Glucose media for 5 minutes. The cilia beats were recorded from the same areas examined prior to the ethanol addition. The movies were analyzed by counting the ciliary beating frequency for both the control and the ethanol-treated tissue.

### *In-vivo* ethanol treatment

Two-month-old Wistar rats were received from Harlan Laboratories Company and housed in the Department of Laboratory Animal Resources at the University of Toledo. All animals were kept in standard plastic tube cage with free access to food and water. The animal room’s temperature and relative humidity was maintained at 25 °C and 50%, respectively with a 12-hour light/dark cycle. The body weight was monitored daily for the rats under treatment.

The animals were divided into two different groups. The control group, totaling six rats, was treated with water by oral gavage for seven days. The rats were deprived of food for two hours prior to oral gavage. The treatment group of six rats were given 95% ethanol, the equivalent of 190-proof by oral gavage. The dose of ethanol is 6 g per kg of body weight divided by the density of ethanol and diluted in deionized water to the total volume of 3 ml.

### Immunofluorescence microscopy

The brain sections were fixed in a phosphate buffered saline (PBS) solution containing 4% paraformaldehyde (PFA) (Electron Microscopy Science Lab, Inc.) and 2% sucrose (Sigma, Inc.) for 10 minutes. The brain slices were incubated in a solution of 0.1% Triton-X (Fisher Scientific, Inc.) in 1X PBS for 5 minutes. Mouse primary antibodies, anti-acetylated α-tubulin (Sigma, Inc.), anti-hydin (Novus Biologicals, Inc.) or anti-polycystin 2 (Santa Cruz Biotechnology, Inc.) were used at a dilution of 1:5,000, 1:30, and 1:250, respectively in a 10% FBS in 1X PBS solution for one hour at room temperature (RT) or overnight at 4 °C. The brain slices were then incubated in the secondary antibodies, fluorescein anti-mouse IgG or texas-red anti-rabbit IgG (Vector Labs, Inc.), at a dilution of 1:500 in a 10% FBS in 1X PBS solution for 1 hour at RT. Before observation under a fluorescent microscope, the sections were counterstained with 4′,6-diamidino-2-phenylindole (DAPI) (Vector Labs, Inc.) for 5 minutes to stain the nucleus/DNA. To minimize photobleaching, the sections were imaged immediately with the minimum exposure time possible.

## Statistics

All the images and movies were captured and analyzed using Metamorph software. All quantitative data were displayed as mean ± SEM. Statistical analysis using student t-test was performed to compare the effect of ethanol on the dynamics of ependymal cilia within the ventricles between ethanol-treated and control groups. All statistical results were considered significant at a significance level of p < 0.05 and denoted by an asterisk (*).

### Data availability statement

All data generated or analyzed during this study are included in this published article (and its Supplementary Information files).

## Electronic supplementary material

Supplementary Information

Supplementary Movie 1

Supplementary Movie 2

Supplementary Movie 3

Supplementary Movie 4

Supplementary Movie 5

Supplementary Movie 6

Supplementary Movie 7

Supplementary Movie 8

Supplementary Movie 9

Supplementary Movie 10

Supplementary Movie 11

Supplementary Movie 12

Supplementary Movie 13

Supplementary Movie 14
