# Supplementary material for: Alcohol consumption impairs the ependymal cilia motility in the brain ventricles
Source: Sci Rep. 2017 Oct 20;7:13652. doi: 10.1038/s41598-017-13947-3 (PMC5651853; doi:10.1038/s41598-017-13947-3)

Alcohol consumption impairs the ependymal cilia motility in the brain ventricles

Al Omran Alzahra J. [1], Saternos Hannah C. [1], Althobaiti Yusuf S. [2], Wisner Alexander[1], Sari Youssef[1], Nauli Surya M. [3], and AbouAlaiwi Wissam A[1].

Movie Legends

Movie 1. Type I ependymal cilia has a minimum beating frequency of 60 Hz and a maximum beating angle of 90°. Type I ependymal cilia beating characteristics are recorded. Ependymal cells in the brain lateral ventricle are captured at 200 frames per second.

Movie 2. Type II ependymal cilia has a beating frequency between 30 and 60 Hz and a beating angle between 90° and 135°. Type II ependymal cilia beating characteristics are recorded. Ependymal cells in the lateral ventricle are captured at 200 frames per second.

Movie 3. Type III ependymal cells has a maximum beating frequency of 30 Hz and a minimum beating angle of 135°. Type III ependymal cilia beating characteristics are recorded. Ependymal cells in the lateral ventricle are captured at 200 frames per second.

Movie 4. Type I ependymal cilia beating decreased following *ex vivo* ethanol treatment. The brain slice was pre-treated with 0.25% ethanol and type I ependymal cilia beating in the brain lateral ventricle were captured at 200 frames per second.

Movie 5. Type II ependymal cilia beating decreased following *ex vivo* ethanol treatment.

The brain slice was pre-treated with 0.25% ethanol and type II ependymal cilia beating in the brain lateral ventricle were captured at 200 frames per second.

Movie 6. Type III ependymal cilia beating decreased following *ex vivo* ethanol treatment. The brain slice was pre-treated with 0.25% ethanol and type III ependymal cilia beating in the brain lateral ventricle were captured at 200 frames per second.

Movie 7. Type I ependymal cilia has a minimum beating frequency of 60 Hz and a maximum beating angle of 90° in the rat brain lateral ventricle. Type I ependymal cilia beating characteristics are recorded. The control rat group was treated with water by oral gavage for seven days. Type I ependymal cilia beating in the brain lateral ventricle are captured at 200 frames per second.

Movie 8. Type I ependymal cilia beating in the rat brain lateral ventricle decreased following *in vivo* ethanol treatment. The treatment rat group was given 95% ethanol by oral gavage at 6g per kg. Type I ependymal cilia beating in the brain lateral ventricle are captured at 200 frames per second.

Movie 9. Type II ependymal cilia has a beating frequency between 30 and 60 Hz and a beating angle between 90° and 135° in the rat brain lateral ventricle. The control rat group was treated with water by oral gavage for seven days. Type II ependymal cilia beating characteristics are recorded. Type I ependymal cilia beating in the brain lateral ventricle are captured at 200 frames per second.

Movie 10. Type II ependymal cilia beating in the rat brain lateral ventricle decreased following *in vivo* ethanol treatment. The treatment rat group was given 95% ethanol by oral gavage at 6g per kg. Type II ependymal cilia beating in the brain lateral ventricle are captured at 200 frames per second.

Movie 11. Type I ependymal cilia has a minimum beating frequency of 60 Hz and a maximum beating angle of 90° in the rat brain third ventricle. Type I ependymal cilia beating characteristics are recorded. The control rat group was treated with water by oral gavage for seven days. Type I ependymal cilia beating in the brain third ventricle are captured at 200 frames per second.

Movie 12. Type I ependymal cilia beating in the rat brain third ventricle decreased following *in vivo* ethanol treatment. The treatment rat group was given 95% ethanol by oral gavage at 6g per kg. Type I ependymal cilia beating in the brain third ventricle are captured at 200 frames per second.

Movie 13. Type II ependymal cilia has a beating frequency between 30 and 60 Hz and a beating angle between 90° and 135° in the rat brain third ventricle. The control rat group was treated with water by oral gavage for seven days. Type II ependymal cilia beating characteristics are recorded. Type I ependymal cilia beating in the brain third ventricle are captured at 200 frames per second.

Movie 14. Type II ependymal cilia beating in the rat brain third ventricle decreased following *in vivo* ethanol treatment. The treatment rat group was given 95% ethanol by oral gavage at 6g per kg. Type II ependymal cilia beating in the brain third ventricle are captured at 200 frames per second.

Supplementary Figure Legends

Supplementary Figure S1. Ependymal cilia in the mouse brain line the lateral and third ventricles. Shown here are ependymal cells from, **a.** the lateral and, **b.** the third ventricle of a mouse brain. The brain sections were stained with acetylated-α-tubulin, a ciliary marker; hydin, an axonemal protein and counter-stained with DAPI, a nucleus marker. Individual differential interference contrast (**top left panel**), and fluorescence (**other panels**) images shown are captured at low magnification (10x). Bar=30 μm.

Supplementary Figure S2. Ciliary structural and functional proteins are expressed in ependymal cilia of mouse brain. Shown here are high-resolution fluorescent microscopy images of ependymal cells from the lateral ventricle of a mouse brain. **a.** The brain section was stained with acetylated-α-tubulin, a ciliary marker, and counter-stained with DAPI, a nucleus marker. **b.** The brain section was stained with acetylated-α-tubulin, a ciliary marker, hydin, a central axonemal marker and counter-stained with DAPI, a nucleus marker. **c.** The brain section was stained with acetylated-α-tubulin, a ciliary marker, polycystin-2, a mechanosensory ciliary protein and counter-stained with DAPI, the nuclear marker. The images were captured at 60, 100 and 150x magnifications. Bar=15 μm.

Supplementary Figure S1

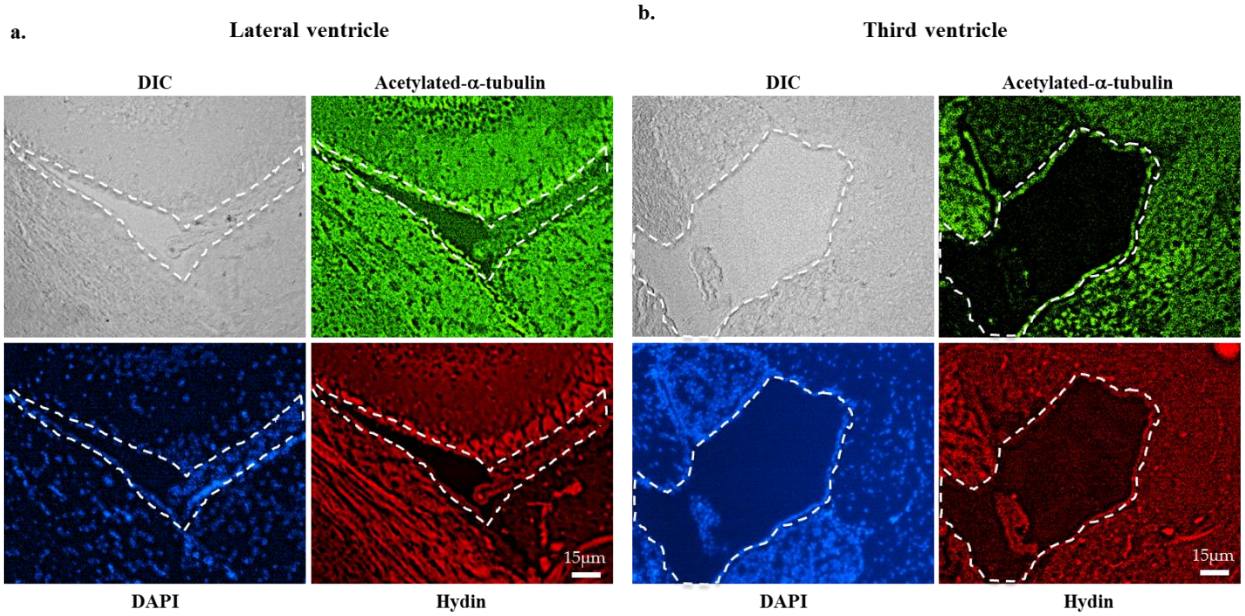

Supplementary Figure S2

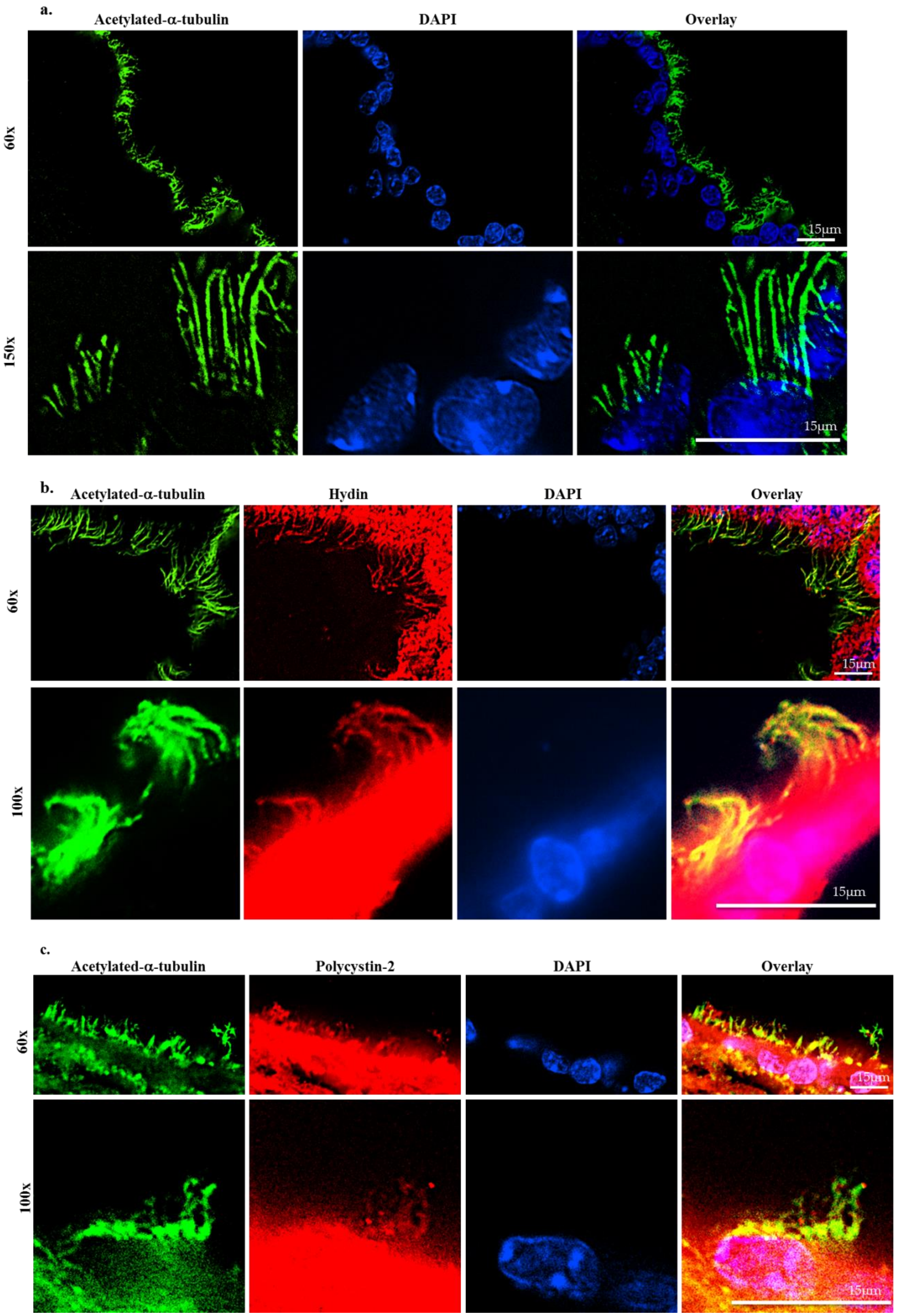

Supplement: Supplementary file 1 — Supplementary Information [file 41598_2017_13947_MOESM1_ESM.pdf]
